# Promoting Parental Loyalty Through Social Responsibility: The Role of Brand Trust and Perceived Value in Chinese Kindergartens

**DOI:** 10.3390/bs15020115

**Published:** 2025-01-23

**Authors:** Xinxin Hao, Chenwei Ma, Min Wu, Lv Yang, Yunxia Liu

**Affiliations:** 1 Institute for Advanced Study in Humanities and Social Sciences, Chengdu University, Chengdu 610106, China; haoxinxin@cdu.edu.cn; 2 School of Public Administration, Sichuan University, Chengdu 610065, China; wu_min@scu.edu.cn; 3 College of Teachers, Chengdu University, Chengdu 610106, China; lvyang@cdu.edu.cn; 4 Chengdu Experimental Foreign Languages School, West Campus, Chengdu 610213, China; 18382173831@163.com

**Keywords:** inclusive kindergarten, educational psychology, early childhood education, corporate social responsibility (CSR), inclusive education

## Abstract

The role of social responsibility in kindergartens is critical for fostering parental loyalty, especially amid declining enrollment rates in China. However, the relationship between kindergarten social responsibility, brand trust, perceived value, and parental loyalty is not well understood. This study investigates the influence of kindergarten social responsibility on parental loyalty, focusing on the mediating roles of brand trust and perceived value. A nationwide survey was conducted, collecting 745 valid responses from parents across 27 provinces in China. Data were analyzed using the PROCESS macro, with mediation effects tested via the bias-corrected nonparametric percentile bootstrap method. The findings reveal that kindergarten social responsibility significantly enhances parental loyalty both directly and indirectly through brand trust and perceived value. Brand trust was identified as the strongest mediator, particularly in non-inclusive kindergartens, where its effect on loyalty was more pronounced. The study also found that parents with higher education levels and higher income tend to have lower perceptions of social responsibility and perceived value, affecting their loyalty. These results suggest that kindergartens must tailor their social responsibility strategies to different parent demographics and kindergarten types to maximize parental loyalty. The study emphasizes the importance of social responsibility in strengthening parental loyalty, with specific implications for inclusive and non-inclusive kindergartens. By understanding the mediating roles of brand trust and perceived value, kindergartens can develop targeted strategies to improve competitiveness and parental engagement.

## 1. Introduction

Early childhood development and education (ECDE) plays a crucial role in the lifelong development of children. Early childhood is a period when all children can engage in meaningful experiential learning and social interaction. It is also a critical stage in their overall development, during which the brain develops rapidly, shaping children’s future learning and social abilities (Mondi et al., 2021; Rao et al., 2023). Children who receive high-quality early childhood education exhibit stronger reading, math, language, and emotion management skills when they enter primary school (Durkin et al., 2022; Yildiz et al., 2023). Additionally, early education plays an important role in promoting social equity. High-quality early education programs can provide equal learning opportunities for children from low-income families and marginalized groups, thereby helping to narrow the educational gap (Hau et al., 2023).

To ensure that all children have access to fair and high-quality early childhood education, countries around the world have increasingly focused on inclusive early childhood education (universal early childhood education) in recent years (Rao et al., 2023). For example, studies have shown that the program significantly improves children’s cognitive and social skills and has long-term effects, including reduced crime rates, increased employment, and higher income levels (Rad et al., 2022; Greenberg, 2023; Schoch et al., 2023). Similarly, Sweden has enacted legislation to ensure that all children aged 1–5 have the right to receive early childhood education, with a focus on creating an inclusive environment that promotes the social participation and development of all children. This inclusive educational environment enhances children’s social skills and emotional management abilities, helping them better integrate into society (Hau et al., 2023).

In China, inclusive preschool education has consistently been a key focus for both society and the government. Through policy support and resource investment, the educational standards for children in rural and impoverished areas have significantly improved (Rao et al., 2023). By the end of 2023, there were 274,000 kindergartens in China, with 236,000 being inclusive kindergartens, accounting for 86.2% of the total. In 2023, China’s gross enrollment rate for preschool children reached 91.1%, marking an increase of 23.6 percentage points from 2013 (Huai, 2024). This achievement also led to the establishment of a preschool education public service system characterized by broad coverage, basic guarantees, and enhanced quality. However, the increased accessibility and equalization of preschool has led to growing concern among parents over security concerns, such as incidents of child abuse, as well as food safety issues and inadequate security measures in some kindergartens. The latest reports of the Chinese Ministry of Education and national media outlets have exposed a number of instances in which the deficiency of child care in kindergartens has triggered massive public outrage and prompted regulation (MOE, 2023). For instance, previous studies have highlighted parents’ dissatisfaction over incidents of teacher misbehavior as well as unsafe learning environments and a lack of accountability from the institution (Banfield et al., 2006; Broeckelman-Post et al., 2016). These issues have led to the decline in enrollment in certain regions, since parents are more concerned with security and confidence when choosing the best kindergarten option for their child (Li et al., 2023). With this low level of trust, knowing how kindergartens can restore trust in parents by implementing social responsibility programs is vital (Dereli & Türk Kurtça, 2023).

Research on CSR in higher education institutions has examined its importance, specifically on student engagement and institution trust (Carvalho & de Oliveira Mota, 2010; El-Kassar et al., 2019; Rasoolimanesh et al., 2023). While there is limited empirical research regarding how CSR influences parent loyalty within kindergarten settings, studies conducted to date on CSR have largely examined its effects on school reputation and the satisfaction of students (Mostafa & Hamieh, 2022; Lv et al., 2022) as well as family involvement in education (Pfajfar et al., 2022), with no exhaustive examination of how trust in brands or the perception of value influence parental loyalty for early childhood educational services. Similar to higher education where students are given more autonomy, parental decision-making in kindergartens is heavily impacted by trust and the perception of value, necessitating a deeper examination of how social responsibility influences loyalty among parents. This research seeks to bridge this gap by investigating both direct and indirect effects of CSR on parental loyalty as well as providing insights regarding both inclusive as well as non-inclusive preschool settings.

In the business field, many studies have shown that Corporate Social Responsibility (CSR) positively impacts customer loyalty. First, CSR enhances customer loyalty by improving corporate image, customer satisfaction, and service quality. Notably, when customers believe that a company is making efforts in legal and economic dimensions, the increase in loyalty is more pronounced. Second, CSR further strengthens customer loyalty by promoting customer citizenship behavior, enhancing trust, and fostering a sense of belonging (E. Park & Kim, 2019; Ahn et al., 2021; Islam et al., 2021).

Existing studies have also explored the role of CSR in education on family and student loyalty. When the CSR activities of educational institutions align with students’ values and expectations, they effectively enhance student loyalty and family involvement in school education. Moreover, when students perceive that a school is effectively fulfilling its social responsibilities, they exhibit higher levels of loyalty (El-Kassar et al., 2019; Mostafa & Hamieh, 2022).

Regarding the impact of kindergarten social responsibility on parental loyalty, some studies have hypothesized a positive correlation between school reputation, parent satisfaction, and parent loyalty (Badri & Mohaidat, 2014; Lv et al., 2022; Dereli & Türk Kurtça, 2023). However, there is still a lack of empirical research on the relationship between kindergarten social responsibility and parental loyalty, highlighting the need for further empirical studies in the field of preschool education to explore the impact of CSR on parental loyalty. Given the importance of this issue and the existing research gap, this study aims to explore how kindergartens can enhance parental loyalty by increasing parents’ trust and perceived value when fulfilling their social responsibilities. The study will analyze the relationship between kindergarten social responsibility and parental loyalty and investigate the external pressures affecting early childhood education institutions and the factors influencing their level of social responsibility.

## 2. Theoretical Framework

The kindergarten categories used in this study have been defined as follows. Publicly Supported Kindergartens (PSKs) are government-supported institutions that ensure wide accessibility to early childhood education. PSKs receive funding and policy support from their state, which enables them to offer low-cost education with effective supervision while upholding quality standards. Privately funded kindergartens operate without government subsidies and rely solely on tuition fees to survive. Each kindergarten may differ in regard to its quality, price, and services offered—often distinguished by using branding, curriculum options, and investments in facilities to attract parents. Universal Preschool Education (UPE) is a policy framework intended to promote access to early education without specifying one type of kindergarten. This policy framework serves as a guideline for expanding, regulating, and making accessible preschool education across regions. Institutional inclusion refers to support from government as well as accessibility while inclusive curriculum refers to accessibility as well as government support; inclusion-based curriculum refers to teaching techniques designed to promote equality and inclusivity during learning experiences.

### 2.1. Development of ECE in China

Early childhood education in China is designed for children aged 3–6. To ensure that more children have access to high-quality, low-cost preschool education services, the Chinese government has actively promoted the development of inclusive kindergartens. These kindergartens are characterized by fairness, public welfare, reasonable fees, and guaranteed education quality, aiming to address the issues of ‘difficulty in admission’ and the ‘high cost of admission’ in preschool education (Lv et al., 2022).

The development of inclusive kindergartens in China has gone through several key stages. In 2010, the Chinese government first introduced the concept of ‘inclusive preschool education’ in the ‘Outline of the National Medium- and Long-Term Education Reform and Development Plan (2010–2020)’, and subsequently promoted the expansion of inclusive kindergartens through various policy measures. According to statistics from the Ministry of Education of China, by 2023, the number of kindergartens in the country had reached 274,000, of which 236,000 were inclusive kindergartens, representing a coverage rate of 86.13%, significantly improving the accessibility of preschool education (MOE, 2023).

Despite the rapid development of inclusive kindergartens in China, frequent incidents in kindergartens have led to a crisis of confidence among parents regarding the quality of these institutions (Rad et al., 2022). At the same time, China’s total fertility rate has continued to decline. The decline in fertility has led to a significant decrease in the number of children entering kindergartens, causing many institutions to face operational difficulties or even bankruptcy due to insufficient enrollment. This situation has posed significant challenges to the field of preschool education. The increase in the number of kindergartens, coupled with a disregard for social responsibility, has influenced parents’ choices. Consequently, how to attract and maintain the loyalty of parents to kindergartens has become an urgent issue that these institutions need to address.

### 2.2. Corporate Social Responsibility and Customer Loyalty

Many factors influence customer loyalty (Mittal et al., 2023). With the growing emphasis on social responsibility, CSR has come to be recognized as a significant factor affecting customer loyalty (Mann & Islam, 2015; Mitnick et al., 2023). The implementation of CSR impacts customer loyalty across multiple dimensions. CSR activities can enhance a company’s image and reputation, thereby increasing customer trust and satisfaction, which in turn improves customer loyalty (Lu et al., 2020). When CSR is viewed as a multidimensional construct, its various dimensions may have different effects on consumer loyalty. For example, charitable activities, environmental protection, respect for consumers, and respect for employees all positively impact customer loyalty (Louis et al., 2019). Additionally, CSR influences customer loyalty indirectly through brand image, enhancing customer trust, and improving customer satisfaction. When consumers perceive a brand as legally and morally responsible, they are more likely to see it as trustworthy and reputable. This perception strengthens customer loyalty by enhancing both the functional and symbolic image of the company (Fatma & Khan, 2023). Cole (2017) found that when companies establish a trustworthy image through transparent and ethical behavior, customers are more likely to increase their trust in the company and are more inclined to purchase its products or services in the long term.

Active CSR activities, such as environmental protection and social welfare, can significantly improve customer satisfaction, which is the foundation of customer loyalty. When customers are satisfied with a company’s socially responsible behavior, their loyalty increases accordingly (Ahmad et al., 2023). In the service industry, the impact of improving CSR on company performance is significantly greater than in other industries. This may be because the service industry interacts more directly with consumers, making them more sensitive to a company’s ethical and social responsibility performance (Casado-Díaz et al., 2014).

### 2.3. Theoretical Perspective

Existing studies have found that social responsibility not only directly affects customer loyalty but also indirectly enhances it by improving variables such as customer trust in the brand and perceived value. First, it is evident that CSR has a positive impact on brand trust. Many studies have shown that CSR can directly and indirectly improve brand image and brand trust. When consumers perceive that a company is fulfilling its social responsibilities, they are more likely to form a positive brand image of the company, thereby further enhancing brand loyalty (He & Lai, 2014; Lu et al., 2020). Wei et al. (2020) demonstrated that customer loyalty can be effectively improved by increasing customer satisfaction and trust. Although CSR generally has a positive impact on brand trust, its effect can be influenced by factors such as product type, type of CSR activity, and individual differences among consumers (Jin et al., 2017). Abd-El-Salam (2021) further found that CSR directly affects brand image and brand trust, and indirectly influences brand loyalty and purchase intention through these variables, emphasizing the mediating role of brand trust between social responsibility and brand loyalty.

The relationship between CSR and perceived value can be analyzed from multiple dimensions. First, the implementation of social responsibility can enhance consumers’ emotional value. When consumers believe that a company is taking on more social responsibility, they feel respected and valued (Green & Peloza, 2011). Second, from a social value perspective, consumers are more likely to choose companies that actively engage in social responsibility, driven not only by moral considerations but also by a recognition of the brand’s social image (Han et al., 2020). Third, from a functional value perspective, consumers view companies that participate in social responsibility activities as more reliable and trustworthy in providing products and services. This sense of trust can translate into higher product satisfaction and loyalty (Bello et al., 2021).

In the field of education, existing studies have also explored the direct and indirect effects of CSR on family and student loyalty. By formulating and implementing social responsibility policies, educational institutions can encourage students to practice values in their daily learning and life, which not only helps to cultivate a sense of responsibility and empathy but also lays the foundation for their active participation in society in the future (Greenberg, 2023). Yang et al. (2023) successfully improved students’ academic performance and increased family involvement in school education by establishing a CSR model that included specific attributes and utilized elements such as teamwork, leadership, and action plans.

El-Kassar et al. (2019) found that CSR not only directly affects student loyalty but also indirectly influences it through school-student identity. Therefore, educational institutions can enhance students’ sense of belonging and loyalty by strengthening their social responsibility. While some studies have hypothesized the relationship between kindergarten social responsibility and parental loyalty—such as Mahmud et al. (2011), who showed that parental perceived value may indirectly affect loyalty by influencing parental satisfaction and trust—there is still no empirical research specifically examining the relationship between kindergarten social responsibility and parental loyalty.

Trust is widely acknowledged as an essential factor in consumer and institutional engagement (Barlas et al., 2023). Trust can be built both directly through direct experience (satisfaction) and indirectly via external confirmation (reputation). In service-oriented industries like education institutions, trust forms through direct experience (satisfaction) as well as external confirmation (reputation). Parents’ satisfaction is measured through their experiences at their child’s kindergarten, in terms of teaching, safety, and the responsiveness to any issues that may arise (Badri & Mohaidat, 2014). Reputation refers to the opinion held by an entire community about an individual or institution, and is affected by peer pressure or public opinion as well as status issues such as funding (Rasoolimanesh et al., 2023). Studies have demonstrated that consumers often develop trust based on both satisfaction with one’s life and reputation (J. Park et al., 2014; Servera-Francés & Piqueras-Tomás, 2019); as these elements reduce uncertainty and encourage ongoing commitment, evaluating trust according to these factors is consistent with behavioral science and consumer research theories.

In light of the continuous decline of children enrolled in kindergartens in China, kindergartens are attempting to improve parental loyalty through various methods to maintain their current scale of operation. Although most inclusive kindergartens receive government subsidies, these are not sufficient to fully address the pressures brought by the declining population. Non-inclusive kindergartens face even greater operational challenges due to the lack of government subsidies. Therefore, to promote the sustainable development of kindergartens and improve the overall quality of education, this paper aims to address three research questions: (a) How does kindergarten social responsibility influence parental loyalty, and what role do brand trust and perceived value play in this relationship? (b) Do brand trust and perceived value mediate the relationship between kindergarten social responsibility and parental loyalty? And, (c) How do these relationships differ between inclusive and non-inclusive kindergartens?

## 3. Research Hypotheses

### 3.1. Kindergarten Social Responsibility and Parent Loyalty

Corporate social responsibility (CSR) disclosure and behavior serve as positive signals that can earn the recognition of stakeholders and guide them to support the development of the enterprise. CSR disclosure and behavior address the concerns of stakeholders, such as partners and government agencies, regarding the process of corporate social value creation, and help to strengthen alliances with external stakeholders. CSR helps to improve customer loyalty (E. Park & Kim, 2019; Zhang, 2022; Leclercq-Machado et al., 2022; Ahmad et al., 2023), with the following key influence paths: first, by enhancing consumers’ positive evaluation of the company, leading to a stronger willingness to purchase the company’s products, which manifests as higher behavioral loyalty; and second, by strengthening customers’ psychological identification with the company, resulting in higher attitudinal loyalty.

The research by Barlas et al. (2023) shows that companies that focus on CSR can create a positive image among customers, thereby enhancing customer loyalty. Zhang (2022) found that the COVID-19 pandemic further highlighted the importance of CSR in maintaining customer loyalty and corporate reputation. The study by Leclercq-Machado et al. (2022) also demonstrated that CSR disclosure and behavior can improve customer loyalty. They noted that the results of their study could serve as a reference for other industries and related sectors in different countries to explore the relationship between social responsibility and customer loyalty. Based on this, the current study will explore the impact of the social responsibility of preschool education institutions on the loyalty of parents, who are the main stakeholders in the preschool education industry, and propose the following hypothesis:

**Hypothesis** **1.** *Kindergarten social responsibility helps enhance parent loyalty.*

### 3.2. Kindergarten Social Responsibility and Brand Trust

Actively fulfilling social responsibilities is an effective way for companies to build brand trust. Numerous studies have confirmed the positive relationship between corporate social responsibility (CSR) behavior and customer trust in brands (Pivato et al., 2008; Vlachos et al., 2009; J. Park et al., 2014). On one hand, responsible corporate behavior is perceived as altruistic, leading consumers to view the company as ethical and trustworthy, thereby reducing uncertainty during the purchasing process. On the other hand, CSR behavior also communicates the company’s values to customers. By incorporating ethical principles and values into strategic decision-making, a company can enhance the trust of all stakeholders, including consumers (Azmat & Ha, 2013). For example, Cheung and Pok (2019) pointed out that organizations perceived by consumers as responsible to the public tend to enjoy a higher degree of trust among stakeholders, including consumers. Similarly, Liu et al. (2021) found that one of the most important effects of companies actively assuming social responsibilities is earning customer trust. For preschool education institutions, demonstrating a commitment to social responsibility helps to gain parents’ trust in these institutions and enhances their trust in the brands. Based on this, the study proposes the following hypothesis:

**Hypothesis** **2.** *Kindergarten social responsibility has a positive impact on brand trust.*

### 3.3. Brand Trust and Parent Loyalty

Trust acts as the social glue between enterprises and stakeholders. When partners trust each other, they spend less time and energy protecting their rights and interests (Farid et al., 2020). Le et al. (2021) suggested that establishing good brand trust in the minds of consumers is an effective way to build and maintain lasting customer loyalty. Enterprises with strong brand trust can earn the trust and support of stakeholders. Trust is often considered the core element in maintaining a successful relationship. Due to the risks and uncertainties in the market, the level of trust consumers has in brands has become a crucial factor influencing their purchasing decisions. In a trusting relationship, both parties demonstrate mutual dependence and are willing to make moderate sacrifices. Consumers are confident that a trusted company will act in their favor in the future, leading them to maintain the relationship in both attitude and behavior. Previous studies have shown that trust enhances people’s willingness to maintain relationships and increases customer loyalty.

Villagra et al. (2021) found in their study of corporate social responsibility (CSR) and consumer behavior that when consumers perceive a company as actively fulfilling its social responsibilities, their trust in the company increases. This trust, derived from CSR initiatives, fosters stakeholders’ sustainable purchasing intentions and behaviors toward the company. Similarly, a study by Huo et al. (2022) also identified the mediating effect of brand trust. The results showed that fulfilling social responsibilities positively impacts stakeholder loyalty, with this effect being mediated by brand trust. Therefore, considering the positive impact of brand trust on stakeholder loyalty and its mediating role between social responsibility fulfillment and stakeholder loyalty, the study proposes the following hypotheses:

**Hypothesis** **3.** *Brand trust has a positive impact on parent loyalty.*

**Hypothesis** **4.** *Brand trust mediates the relationship between kindergarten social responsibility and parent loyalty.*

### 3.4. Kindergarten Social Responsibility and Parents’ Perceived Value

Consumer perceived value is the overall assessment of what people receive in relation to what they pay when obtaining a product or service. It is an important variable for measuring consumers’ evaluations of corporate brands and their products. Research by Joo et al. (2019) shows that genuine social responsibility actions by a company can significantly impact its reputation, such as transparency improvement programs in social responsibility projects, which help to strengthen relationships between companies and consumers and enhance consumers’ evaluations of the company. Jalilvand et al. (2017) pointed out that companies can attract investors and increase consumer trust and recommendations through positive social responsibility behaviors.

The research results of previous studies indicate that corporate social responsibility serves as an important signal of a responsible company, expressing the company’s inherent values and virtues (Echezona, 2024), and can enhance customers’ perceptions and evaluations of the company’s products (Joo et al., 2019; S. Lee et al., 2020). Similarly, other studies have shown that an organization’s social responsibility activities promote stakeholders’ positive evaluations of the organization’s products or services (Joo et al., 2019; Fatma & Khan, 2023) and motivate them to share information about the brand (S. Lee et al., 2020; Araújo et al., 2023). For preschool education institutions, their image of fulfilling social responsibilities may encourage parents to positively evaluate them and enhance parents’ perceived value of these institutions. Based on this, the study proposes the following hypothesis:

**Hypothesis** **5.** *Kindergarten social responsibility has a positive impact on parents’ perceived value.*

### 3.5. Parents’ Perceived Value and Parent Loyalty

Existing research has established a close connection between consumer perceived value and brand loyalty. Konuk (2018) and other scholars believe that perceived value helps consumers to distinguish brands among competing companies and provides them with reasons to buy. Research by Solikhah and Amelia (2023) shows that consumer perceived value has a positive impact on brand loyalty. When consumers perceive a brand as offering better quality, they attribute a higher value to it, which in turn influences their decision to repurchase the same brand. Muskat et al. (2019) also argue that in terms of service, perceived value is a key factor in strengthening the perceived superiority of a brand, ultimately enabling it to surpass its competitors. Similar studies also point out that high-quality brands enhance consumers’ self-esteem, thereby encouraging repeat purchases and reducing the likelihood of switching to other brands (Islam et al., 2021; Lacap et al., 2021; Fatma & Khan, 2023). Furthermore, existing studies have identified the mediating role of consumer perceived value between corporate social responsibility (CSR) and consumer loyalty (Leclercq-Machado et al., 2022; Fatma & Khan, 2023; Ahmad et al., 2023). When consumers recognize the long-term efforts of companies in fulfilling their social responsibilities, it improves stakeholders’ evaluations of the company. As these positive perceptions become more readily accessible in memory, the predicted loyalty also increases. For example, the research by Safeer and Liu (2023) highlighted that genuine CSR activities positively impact brand loyalty by improving brand trust and perceived value. The study by Kim et al. (2020) emphasized that consumers’ perception of quality fosters positive emotions, which in turn enhances loyalty.

Given this, it can be considered that stakeholders’ perceived value has a positive impact on loyalty, and perceived value acts as a mediating variable that connects the positive relationship between organizational social responsibility and stakeholder loyalty. Therefore, this study proposes the following hypotheses:

**Hypothesis** **6.** *Parent perceived value has a positive impact on parent loyalty.*

**Hypothesis** **7.** *Parents’ perceived value mediates the relationship between kindergarten social responsibility and parent loyalty.*

**Figure 1:**
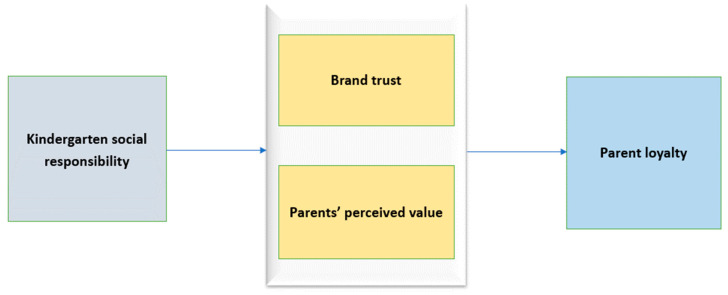
Conceptual model and research hypotheses (source: developed by the authors).

The conceptual model represents all major constructs and their relationship (Figure 1).

## 4. Research Methodology

### 4.1. Measurement of Variables

Kindergarten social responsibility is measured through four dimensions: responsibility management, customer responsibility, public welfare responsibility, and organizational responsibility. The kindergarten social responsibility assessment scale constructed by Lv et al. (2022) was used as a reference, along with the social responsibility assessment standards for BRICS enterprises developed by Arrive and Feng (2018). Additionally, the ISO 26000 international standard guide for social responsibility and the consumer corporate social responsibility recognition scale developed by Wang and Xu (2017) were incorporated.

Brand trust was measured through parent satisfaction and kindergarten reputation (Le et al., 2021; Villagra et al., 2021). Parents’ perceived value was assessed using the two dimensions of value for money and quality (Rad et al., 2022). For the measurement of parental loyalty, we referred to the attitudinal loyalty and behavioral loyalty dimensions developed by Y.-C. Lee et al. (2016). The specific scale is presented in Table 1. To maintain conceptual coherence, we classify our variables within a hierarchical framework in which higher-level constructs serve as broad conceptual domains while sub-dimensions provide specific elements within them. Higher-level constructs include social responsibility, brand trust, parents perceived value, and parent loyalty; sub-dimensions represent specific aspects of these higher-level constructs.

While most constructs were evaluated with multi-item scales to improve the reliability of tests, some lower-level concepts (e.g., particular attributes of reputation and social responsibility) were evaluated using single-item measures instead. This decision was informed by prior research which has demonstrated that single-item measures can provide reliable responses while reducing fatigue from surveys; multi-item measures were used instead for more general variables like trust between parents and brand loyalty that require deeper evaluations.

In this study, trust is measured using two indicators—parent satisfaction and kindergarten reputation—as two objective and socially constructed events. Parent satisfaction provides direct measures of trust between kindergartener interactions and external perceptions and peer influence (Lv et al., 2022); reputation captures this notion more indirectly among all class members (Badri & Mohaidat, 2014). This method aligns well with previous studies regarding trust development within services and education industries, where both elements play key roles in building this across institutions and with stakeholders.

To ensure the integrity of our methodology, we distinguish between formative and reflective concepts within our framework. The trust of brands and their perceived worth as well as parental trust are considered reflective constructs since their indicators reveal what lies at their core. Any changes within the latent variable can result in uniform variation among its indicators, thus validating confirmatory factor analyses (CFA). Social responsibility can be defined as an abstract formal concept because its sub-dimensions (responsibility management, customer responsibilities, public welfare responsibility, and organizational responsibility) define its term instead of simply representing it interchangeably. Modifications in one dimension do not automatically result in similar modifications elsewhere.

### 4.2. Sampling and Data Collection

This study used a stratified random sampling method to ensure that its sample was representative across various regions and socioeconomic contexts in China. The sample consisted of parents of children attending both privately and publicly funded kindergartens in 27 provinces as well as 124 cities, stratified according to geographical distribution (eastern central, central west, eastern regions) as well as type of kindergarten; stratification methods focused on variations in parental attitudes between kindergarten types. This strategy increased generalizability while simultaneously creating an adequate representation of demographic characteristics as well as institution characteristics within its sample population.

To facilitate easier understanding, this study categorizes kindergartens according to their funding structures and accessibility needs. Publicly supported kindergartens (N = 518) are government-subsidized institutions which ensure affordability and compliance with national education policies, providing wide access to early childhood education. Privately funded kindergartens (N = 227) operate independent of the state and primarily rely on tuition and investments for funding their operations. All kindergartens (N = 745) represent all schools across both types of funding sources—private as well as public institutions are included to ensure uniformity across classification.

This study was conducted nationwide, using a combination of online and offline questionnaire surveys. Questionnaires were distributed to parents of children in preschool education institutions across the country. A total of 820 questionnaires were distributed, with 782 collected, resulting in a collection rate of 95.3%. After eliminating invalid and incomplete questionnaires, 745 were deemed valid, achieving an efficiency rate of 95.2%. The sample included participants from 27 provinces and 124 cities, with 42.1% from cities in the eastern and coastal areas, and 40.9% from cities in the central and northern regions. The regional distribution of the survey participants was relatively even. The data were collected from 20 August 2024 to 16 September 2024.

Each item of the questionnaire was evaluated by using an a 5-point Likert scale that ranges between 1- (strongly disagree) through 5 (strongly agree). The scale was selected to measure the varying levels of parents’ perceptions about kindergarten, social responsibility, and brand trust, as well as perceived worth and loyalty.

### 4.3. Statistical Analysis

Before conducting hypothesis testing, this study first used Harman (1968)’s single-factor test to check for common method bias in the variables and samples. Secondly, exploratory factor analysis and confirmatory factor analysis were conducted on the scale used in this study to test whether the indicators were structurally consistent, reliable, and valid. To explore the relationships between kindergarten social responsibility, brand trust, parent perceived value, and parent loyalty, the study used SPSS 29.0 combined with the PROCESS program developed by Hayes (2022) to test the mediation model. In this study, Hayes PROCESS macro (Model 6) was utilized as the mediation analysis software due to its capability of assessing indirect effects using bootstrapping, which is a nonparametric technique which offers reliable confidence intervals while avoiding normality assumptions in contrast to more traditional tests like Baron and Kenny (1986) or Sobel tests (Hayes, 2022). PROCESS is widely utilized in behavioral science and consumer psychology due to its capacity for the simultaneous assessment of multiple mediation pathways while controlling for covariates, making it particularly helpful when working with smaller samples. When compared with structural equation models (SEM), PROCESS excels by targeting specific mediation factors instead of general latent construct relations. Model 6 was selected due to its capacity to test for sequential mediation effects, where trust in a brand and perceptions of value create an indirect causal chain in relation to CSR and parental loyalty. The bias-corrected nonparametric percentile bootstrap method was employed to assess the significance of the mediation effect, and Model 6 provided by Hayes was selected for analysis.

## 5. Results

### 5.1. Common Method Bias Test

Common method bias refers to the artificial covariation between the predictor variable and the criterion variable caused by factors such as the same data source, the same measurement environment, the project context, and the characteristics of the project itself. It is a systematic error that can cause significant distortion in research results and potentially lead to misleading conclusions (Kock et al., 2021). There are various statistical tests and control methods for common method bias. One commonly used method is the Harman single-factor test, which involves using confirmatory factor analysis to extract a common factor from the variables involved. The data results showed that the data and the model did not fit effectively (χ^2^/df = 4.9, NFI = 0.87, IFI = 0.89, CFI = 0.89, RMSEA = 0.07). This indicates that, although the research used a questionnaire survey method and all data were collected from preschool education institutions, there is no serious common method variance, and it does not affect the accuracy of the research results.

### 5.2. Reliability and Validity Test

To ensure the reliability of the questionnaire, the results needed to be tested for reliability. This study used SPSS 29.0 to analyze the reliability of the ‘Parent evaluation questionnaire for preschool education institutions’. The results showed that the Cronbach’s alpha coefficient for the social responsibility dimension was 0.816, for the brand trust dimension 0.948, for the parent perceived value dimension 0.718, and for the parent loyalty dimension 0.720. All coefficients reached an acceptable level and passed the reliability test.

The validity test primarily examines the content validity and structural validity of the questionnaire (Solikhah & Amelia, 2023). For content validity, since the scale in this study was designed based on the literature analysis, expert-assisted revisions, and adaptation from relevant mature scales, it possesses good content validity. For structural validity, this study employed the KMO and Bartlett’s sphericity tests, along with factor analysis. Before conducting factor analysis, the KMO and Bartlett’s sphericity tests were performed. The results showed that the KMO value for the scale was 0.932, and the significance level for Bartlett’s sphericity test was 0.000, indicating that factor analysis was appropriate. Subsequently, AMOS 23.0 software was used to construct a structural equation model for confirmatory factor analysis.

Confirmatory factor analysis (CFA) results indicate an extremely good fit to the model that meets the generally accepted thresholds. The ratio kh2/df (2.863) lies within the recommended range for CFA of 3.0 which suggests a good balance between complexity of model and data fit. GFI = 0.937 exceeds the 0.90 benchmark, signifying that the model hypothesized can capture observed variability. The Root Mean Square Residual (RMSR = 0.025) and Root Mean Square Errors of Approximation (RMSEA = 0.050) values were both less than 0.06; this indicates minimal residual error and near approximation to real world scenarios. Additionally, incremental fit indexes including Normed Fit Index (NFI = 0.924), Tucker–Lewis Index (TLI = 0.941) and Comparative Fit Index (CFI = 0.949) revealed strong accuracy for this model. These results confirm its theoretical framework while showing validity for its measure model, validating hypothesis testing as well as the overall methodological reliability of the approach in study. The analysis results for each scale are as follows (Table 2).

In Table 2, the model fit indices of the scale in the ’Preschool Education Institution Parent Evaluation Questionnaire’ indicate a good fit. Additionally, the standardized loading values for each latent variable measurement item are greater than 0.75, and the average variance extracted (AVE) for each factor is greater than 0.5, demonstrating good convergent validity. In summary, the reliability and validity of the scale have been verified.

### 5.3. Basic Statistics of Samples

This study conducted frequency and percentage statistics on the basic information of all kindergartens, including both inclusive and non-inclusive kindergarten samples. The variables analyzed included kinship, education, monthly family income, monthly kindergarten fees, kindergarten class, kindergarten type, kindergarten grade, and location. The specific analysis is presented as follows (Table 3).

Among kindergarten samples, 64.16% of respondents were mothers, highlighting their significant role in early childhood education, with 32.75% being fathers. College graduates made up 48.99% of the parents, showing the importance of higher education in early education concerns. Most families had a monthly income of CNY 10,000 to 30,000, indicating middle-class predominance, and most kindergartens charged CNY 1000 to 3000 monthly. In inclusive kindergartens, 61.2% of respondents were mothers, and 55.4% held undergraduate degrees, with most families earning between CNY 10,000 and 30,000. Non-inclusive kindergartens had 70.9% of respondents as mothers, with lower education levels and incomes between CNY 5000 and 10,000, indicating a slightly lower economic status. Fee structures and class distributions varied slightly, with non-inclusive kindergartens generally charging less and being more common in central and northern regions.

### 5.4. Mean and Standard Deviation of Scores for Each Dimension

The mean and standard deviation of parents’ scores for social responsibility, brand trust, perceived value, and loyalty were compared between public and privately funded kindergartens. The results are presented in Table 4.

Overall, parents rated the fulfillment of social responsibilities by preschool education institutions nationwide at 4.0, reflecting general satisfaction. Publicly supported kindergartens received a slightly higher rating (4.1) compared to privately funded kindergartens (3.9), particularly in responsibility management (4.1 vs. 3.9), customer responsibilities (4.0 vs. 3.7), and public welfare responsibility (4.0 vs. 3.7). However, privately funded kindergartens exhibited greater variance in parental perceptions, suggesting disparities in service consistency. Additionally, significant variability in opinions on class size and admission conditions suggests room for improvement in these areas.

Parents rated brand trust highly, with publicly supported kindergartens scoring 4.2 and privately funded kindergartens 4.1. Despite this, privately funded kindergartens received lower scores for kindergarten reputation and parent satisfaction, indicating unmet expectations in hardware facilities, teaching environment, staff, and teaching quality. In perceived value, publicly supported kindergartens scored higher (4.0) than privately funded kindergartens (3.7), particularly in cost-effectiveness (4.0 vs. 3.7) and quality (3.9 vs. 3.7). This likely contributes to higher parent loyalty to publicly supported kindergartens, with a score of 4.2, compared to 4.1 for privately funded kindergartens, as parents are more likely to continue choosing and recommending them due to greater trust and satisfaction.

### 5.5. Analysis of Differences in Parent Evaluations Based on Different Sample Characteristics

This study employs one-way analysis of variance (ANOVA) to analyze the differences in the mean comparison of social responsibility, brand trust, perceived value, and loyalty toward preschool education institutions among parents with different educational backgrounds, varying monthly incomes, and children attending different kindergartens, grades, and fee levels (Table 5).

As seen in the table above, several factors significantly influence parents’ evaluations of preschool education institutions: Firstly, parents’ evaluations of preschool institutions’ social responsibility decrease with higher educational levels. Master’s degree holders have the lowest ratings (3.85 for social responsibility, 3.81 for perceived value), likely due to higher expectations. Secondly, parents’ income influences their ratings. Lower-income parents (<5000 CNY) rate social responsibility (4.25) and perceived value (4.12) higher than middle-income parents (CNY 10,000–30,000), who give lower ratings (3.87 and 3.77) possibly due to differing expectations. Thirdly, parents of children in inclusive kindergartens rate these institutions higher in social responsibility (F = 9.685, *p* < 0.01) and perceived value (F = 17.955, *p* < 0.001), reflecting higher trust and loyalty. Fourthly, higher-level kindergartens, like municipal first-class, receive the highest ratings in social responsibility (4.10), brand trust (4.31), and loyalty (4.33). Lower-level kindergartens receive lower ratings. Lastly, kindergartens with lower fees (<1000 CNY) receive higher ratings across all measures (social responsibility: 4.14, brand trust: 4.26, perceived value: 4.11, loyalty: 4.22). High-fee kindergartens (>8000 CNY) receive lower ratings, possibly due to concerns over cost-effectiveness.

### 5.6. Relationship Analysis and Mediation Testing of Kindergarten Social Responsibility and Parent Loyalty

To explore the relationships between kindergarten social responsibility, brand trust, parent perceived value, and parent loyalty, this study used the PROCESS program developed by Hayes to test the mediation model. The bias-corrected nonparametric percentile bootstrap method was employed to test the significance of the mediation effect, with Model 6 provided by Hayes selected for analysis.

#### 5.6.1. Correlation Analysis Among Variables

This study first used the Spearman correlation coefficient to test the correlation between variables such as kinship, parent education, family monthly income, kindergarten monthly fees, kindergarten type, kindergarten level, social responsibility, brand trust, parent loyalty, and parent perceived value (Table 6).

As seen in Table 6, there is a significant positive correlation between parents’ evaluations of the social responsibility of preschool education institutions and parents’ perceived value, parent loyalty, and brand trust. Additionally, there is a significant positive correlation between kinship and parents’ perceived value, while a significant negative correlation exists between parents’ education level and family monthly income with parents’ perceived value. Furthermore, there is a significant negative correlation between kindergarten monthly fees, kindergarten type, and kindergarten level with parents’ loyalty and brand trust.

Next, Pearson correlation analysis (Table 7) was used to test the correlation between kindergarten social responsibility, brand trust, parent perceived value, and parent loyalty.

As shown in Table 7, kindergarten social responsibility, brand trust, parent perceived value, and parent loyalty are all significantly positively correlated.

#### 5.6.2. Test of Mediation Effect

The SPSS macro program Process, developed by Hayes, was used to conduct mediation effect analysis. Since the overall descriptive analysis of the variables has shown that kinship, parent education, family income, kindergarten fees, kindergarten grade, and kindergarten type impact kindergarten social responsibility, brand trust, parent perceived value, and parent loyalty, these factors were included as control variables to avoid interference (Table 8).

Therefore, controlling for kinship, parent education, family monthly income, kindergarten fees, kindergarten grade, and kindergarten type, the mediating roles of brand trust and parent perceived value between kindergarten social responsibility and parent loyalty were analyzed. The results showed that kindergarten social responsibility had a direct positive predictive effect on brand trust (β = 0.583, *p* < 0.001) and parent perceived value (β = 0.267, *p* < 0.001). Additionally, brand trust had a direct positive predictive effect on parent perceived value (β = 0.786, *p* < 0.001). When kindergarten social responsibility, brand trust, and parent perceived value were simultaneously used to predict parent loyalty, all three had a significant positive predictive effect on parent loyalty (β = 0.101, *p* < 0.05; β = 0.542, *p* < 0.001; β = 0.178, *p* < 0.001).

The bias-corrected nonparametric percentile bootstrap method was used to further test the mediation effect. The results are presented in Table 9, showing that the mediating effects of brand trust and parent perceived value are significant, with a mediation effect value of 0.084. Firstly, the mediation effect operates through three mediation chains. The first chain, indirect effect 1 (0.432), consists of the following path: kindergarten social responsibility → brand trust → parent loyalty. The bootstrap 95% confidence interval does not include 0, indicating that brand trust has a significant mediating effect. Secondly, the second chain, indirect effect 2 (0.044), follows this path: kindergarten social responsibility → parent perceived value → parent loyalty. The bootstrap 95% confidence interval does not include 0, indicating that parent perceived value has a significant mediating effect between kindergarten social responsibility and parent loyalty. Finally, the third chain, indirect effect 3 (0.075), involves the following path: kindergarten social responsibility → brand trust → parent perceived value → parent loyalty. The bootstrap 95% confidence interval does not include 0, indicating that brand trust and parent perceived value together create a significant chain mediating effect between kindergarten social responsibility and parent loyalty.

As shown in Table 9, the indirect effect 1, consisting of the path kindergarten social responsibility → brand trust → parent loyalty, has the largest effect value of 0.432, accounting for 78.40% of the total indirect effect. Therefore, brand trust plays a major mediating role between kindergarten social responsibility and parent loyalty.

**Figure 2:**
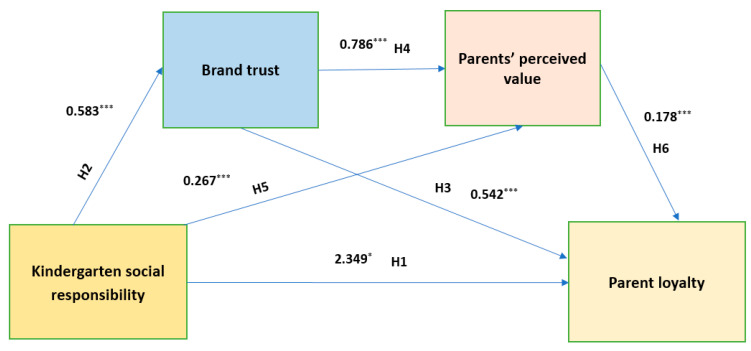
The mediating effect of brand trust and parental perceived value. Note: *, and *** denote statistical significance at the 10%, and 1% levels, respectively. (Source: developed by the author based on the analysis results.).

The specific path through which kindergarten social responsibility influences parent loyalty is illustrated in Figure 2.

### 5.7. Analysis of Heterogeneity of Mediating Effects of Kindergartens of Different Natures

To further verify and compare the mediating role of brand trust in the relationship between the social responsibility of inclusive and non-inclusive kindergartens and parent loyalty, the study used Model 4 provided by Hayes for a heterogeneity analysis (Hayes, 2022). This analysis focused on the mediating effect of brand trust on the relationship between the social responsibility of inclusive and non-inclusive kindergartens and parent loyalty. The analysis was conducted while controlling for variables such as kinship, parent education, family income, kindergarten level, and kindergarten fees.

The regression analysis for the variables related to inclusive kindergartens (Table 10) indicated that the social responsibility of inclusive kindergartens had a direct positive predictive effect on brand trust (β = 0.676, *p* < 0.001). Furthermore, when both the social responsibility of inclusive kindergartens and brand trust were used to predict parent loyalty, both factors had a significant positive predictive effect on parent loyalty (β = 0.104, *p* < 0.05; β = 0.585, *p* < 0.001).

As shown in Table 11, the bias-corrected nonparametric percentile bootstrap method was used to further test the mediation effect (Chen et al., 2023). The results showed that the upper and lower limits of the total effect’s bootstrap 95% confidence interval did not include 0, indicating that the total effect was significant. Similarly, the upper and lower limits of the indirect effect’s bootstrap 95% confidence interval did not include 0, indicating that brand trust played a mediating role between the social responsibility of inclusive kindergartens and parental loyalty, with a mediation effect value of 0.529, accounting for 73.13% of the total effect. Additionally, the upper and lower limits of the direct effect’s bootstrap 95% confidence interval did not include 0, indicating that the direct effect was significant and that brand trust played a partial mediating role between the social responsibility of inclusive kindergartens and parental loyalty.

The regression analysis for the variables related to non-inclusive kindergartens (as shown in Table 12) indicated that the social responsibility of non-inclusive kindergartens has a direct positive predictive effect on brand trust (β = 0.632, *p* < 0.001). Additionally, when both the social responsibility of non-inclusive kindergartens and brand trust were used to predict parent loyalty, both factors had a significant positive predictive effect on parent loyalty (β = 0.124, *p* < 0.05; β = 0.732, *p* < 0.001).

In Table 13, the bias-corrected nonparametric percentile bootstrap method was used to further test the mediation effect (Leclercq-Machado et al., 2022). The results showed that the upper and lower limits of the total effect bootstrap 95% confidence interval did not include 0, and the total effect was significant. The upper and lower limits of the indirect effect bootstrap 95% confidence interval did not include 0, indicating that brand trust played a mediating role between the social responsibility of non-inclusive kindergartens and parent loyalty, and the mediation effect value was 0.573, accounting for 89.63% of the total effect. The upper and lower limits of the direct effect bootstrap 95% confidence interval did not include 0, and the mediation effect value was 0.062, accounting for 10.37% of the total effect.

Brand trust plays an important mediating role between social responsibility and parent loyalty in both inclusive and non-inclusive kindergartens, but there are significant differences in the degree of mediation.

In inclusive kindergartens, both brand trust and social responsibility have a significant impact on parent loyalty. The mediating effect of brand trust accounts for 73.13% of the total effect, indicating that the social responsibility of inclusive kindergartens not only affects parent loyalty directly but also enhances it further by improving brand trust. In non-inclusive kindergartens, the mediating effect of brand trust is even more pronounced, accounting for 89.63% of the total effect. This suggests that in non-inclusive kindergartens, the impact of social responsibility on parent loyalty is primarily achieved through improving brand trust, while the direct effect is relatively smaller. This may reflect the larger disparities in teaching quality, safety management, hardware facilities, and other aspects in non-inclusive kindergartens. Parents may rely more on brand trust to compensate for these differences in order to maintain their loyalty to the kindergarten.

In summary, the mediating role of brand trust differs between inclusive and non-inclusive kindergartens. These differences should be carefully considered in policy-making and kindergarten management to enhance parental loyalty.

## 6. Discussion

### 6.1. Overview of the Insights

This study sought to investigate the role of social responsibility in Chinese kindergartens in encouraging parental loyalty by exploring its dynamics between social responsibility, brand trust, perceived value, and parental loyalty. It provided significant insights into this relationship while emphasizing brand trust’s and perceived value’s mediating roles in relation to parental loyalty. The findings provided valuable insights into this dynamic between kindergarten social responsibility and parental loyalty and provided concrete examples that reinforced this through brand trust and perceived value mediating roles.

Firstly, this study demonstrated how social responsibility can significantly increase parents’ loyalty during kindergarten. These findings are in line with prior corporate studies which demonstrated how corporate social responsibility (CSR) increased customer loyalty by improving image of business, customer satisfaction, and the perceived quality of service (Echezona, 2024). For kindergartens specifically, transparency practices such as safety monitoring and engagement with community are invaluable in building faith between parent and staff; consequently, their payoff can be very encouraging (Barlas et al., 2023).

Secondly, the results showed that trust in brands and perceived value play important roles in mediating the connection between parental responsibility and social loyalty. Brand trust specifically was found to be the most powerful intermediary in schools that do not have an inclusive curriculum, which suggests that the children’s parents who attend these types of institutions are heavily reliant on their faith in the brand of their kindergarten in forming a loyalty. This research is in line with research that has highlighted how important trust is in maintaining loyalty to customers particularly in service-oriented fields such as education (Huo et al., 2022).

Finally, the study revealed distinct differences in the way the social responsibility of parents affects their loyalty in different kinds of kindergartens. In inclusive kindergartens, the social responsibility factor had a greater direct effect on parental loyalty. It was also dependent on brand trust. Contrarily to the non-inclusive schools, the impact of social responsibility on loyalty was much more affected by trust in the brand and had a less direct impact. This suggests that kindergartens that are not inclusive could be required to prioritize building trust in their brand to make up for perceived weaknesses on other fronts, like the availability of resources and support for policy. The study also found the same results regarding the lack of resources and support for policy (Safeer & Liu, 2023).

### 6.2. Impact of Kindergarten Social Responsibility on Parental Loyalty

This study’s findings support the notion that social responsibility in kindergarten positively impacts parental loyalty. Recent incidents at Chinese kindergartens that threaten parental trust may be one reason for lower enrollment rates (Cullaty, 2011). Due to increased public scrutiny following several incidents of neglect, parents are becoming more selective when selecting kindergartens that have good reputations for safety, ethical management, and transparency (Lv et al., 2022). This study supports these trends by showing that social responsibility in the form of robust safety policies and active parental engagement plays an essential part in rebuilding trust and creating long-term parental loyalty (Lv et al., 2022). These findings align with those from wider research conducted both educationally and in business settings, where CSR has been demonstrated to enhance customer loyalty through increasing trust, satisfaction, and perceived value for customers (Fatma & Khan, 2023). Studies of Malaysian private higher education establishments demonstrated the truth of this statement by showing how CSR initiatives positively impacted customer loyalty through building trust and increasing brand reputation (Rasoolimanesh et al., 2023). Similar findings were seen in educational settings located in Batam City, where CSR proved to significantly increase loyalty among university students. Furthermore, CSR research in Malaysia’s retail industry demonstrated its mediation effect of brand trust and image in increasing brand trust (Rasoolimanesh et al., 2023).

Furthermore, the literature shows that CSR initiatives can enhance customer loyalty across industries (Latif et al., 2020; Zhang, 2022; Barlas et al., 2023). Our research has confirmed this fact by showing how such efforts increase consumer perceptions resulting in stronger emotional bonds and lasting commitment to brands. Research carried out within the Egyptian retail industry has demonstrated how CSR initiatives indirectly impact brand loyalty by serving as mediators between brand image and trust (Abd-El-Salam, 2021). This finding is particularly notable when applied to non-inclusive kindergartens. This study revealed that trust in brands was an integral component of both parental and social responsibility, reflecting findings of research focusing on Pakistani consumers which demonstrated how trust and brand loyalty play an integral part in contributing to CSR initiatives and sustainable purchasing plans (Huo et al., 2022).

Comparing these findings to CSR research reveals that positive impacts of social commitment to loyalty do not just apply in one sector, such as education, but can be found throughout a number of industries, such as tourism (S. Lee et al., 2020). This study’s outcomes add to an ever-increasing body of evidence proving that socially responsible behavior is key to building and maintaining loyalty to any business, whether commercial or educational institute. Cross-sectoral implications highlight the strategic importance of CSR as an instrument to build long-term trust among stakeholders. Studies on CSR authenticity demonstrate this through increased trust in brand perception as well as increased loyalty through word of mouth (Markovic et al., 2022).

### 6.3. Differences Between Inclusive and Non-Inclusive Kindergartens

The impact of social responsibility on parental loyalty varies significantly between inclusive and non-inclusive kindergartens. When there are inclusive preschools backed by government support and policies that have more far-reaching security effects, social responsibility may have a more direct effect on parent engagement. Kindergartens play an essential social function by providing children from all backgrounds access to an early preschool education of high quality, regardless of background. Parents tend to form loyalty to an institution based on its adherence to broad societal values. This direct impact of social responsibility on parental loyalty aligns with findings from studies in other sectors (Badri & Mohaidat, 2014), where the presence of strong institutional support often amplifies the effect of CSR initiatives.

As opposed to inclusive kindergartens, non-inclusive kindergartens rely heavily on trust established with parents through brands as mediators. Without access to as much government support or resources, these kindergartens depend heavily on establishing lasting relationships through trust with them and their reputation amongst their stakeholders—most specifically parents. The study revealed the fact that brand trust plays an important role in mediating between parents’ responsibility and social trust in non-inclusive kindergarten environments. Brand trust could be crucial to organizations that compete in a market that is becoming increasingly competitive in which parents pick their provider according to their perceptions of the quality of their services and their reliability (Na et al., 2023). This finding is consistent with research in other industries, where brand trust is often the key factor in customer loyalty, especially when other forms of institutional support are lacking.

Different levels of trust in brands between inclusive and non-inclusive kindergartens may be the reason for differing expectations of parent involvement. Publicly supported kindergartens undergo a systematic external validation, which includes government quality tests, regular inspections, and adherence to national guidelines for education. These certifications give parents peace of mind from these institutions, increasing trust with these establishments. Meanwhile, privately funded kindergartens enjoy greater autonomy without being subject to as many stringent state assessments—trust from parents is more reliant upon trustworthiness, direct experience or referrals from friends, rather than an established reputation of the institution itself. Parents may feel it is more essential for non-inclusive schools to prove their reliability and credibility since they do not receive the same amount of external validation. Trust has become more important as a basis to create loyalty among consumers, especially in non-inclusive settings. These same trends can be noted in the retail and service industries where trust plays a significant element in influencing consumer preferences, even when the signs of standards and security are not immediately apparent (Na et al., 2023).

### 6.4. Mediating Role of Brand Trust and Perceived Value

This study highlighted the importance of facilitating confidence between the brands as well as their customers, the perceived value of social responsibility, and parental loyalty as a way to improve connections between them. Brand trust—which is, the trust of parents in the capacity of the school to provide high-quality education and support—was identified as an important mediator between parents’ involvement with the school and the quality of support and education services that are provided (Na et al., 2023; Tamanna, 2024). Kindergartens that participate in activities that promote the socially responsible behavior of ensuring safety is maintained as well as ensuring transparency and helping to increase the wellbeing of communities and building trust among their patrons and rise the loyalty of parents. The perception of value was a significant mediator. Parents who felt they gained more from kindergarten’s socially responsible policies are more inclined to stay loyal, a finding that is consistent with studies which have revealed trust in brands as well as perceived mediators (Louis et al., 2019; Echezona, 2024).

The importance that these mediators play in the setting of kindergartens is especially evident when comparing them to other sectors. In the retail and higher education, trust in brands is an important factor that increases customer loyalty through reducing perceived risks and improving the perception of reliability for the business. This is particularly relevant in the service-oriented sector, in which the nature of services is a key factor in loyalty. The perception of value, however, is vital in both educational and commercial contexts as it reflects the completeness of the service a consumer receives in relation to the value they are willing to exchange (Mann & Islam, 2015). In the case of kindergartens, this could include how well the educational program is provided and the safety of the facility as well as the overall experience of the child and parents. Research conducted in these areas has revealed similar mediation effects, which show the universality of these ideas in a wide range of scenarios (Hu et al., 2021).

One amazing result of this research was its findings about differences in mediation effects between kindergartens. Brand trust proved an effective mediator both at all-inclusive kindergartens and non-inclusive preschools; its influence was stronger among non-inclusive kindergartens. Families enrolled in non-inclusive kindergartens may rely more heavily on trust due to a lack of support factors like government subsidies or social support systems (Lv et al., 2022). Contrasting with inclusive kindergartens where external support was more prominent, the perception of value played a greater mediating role, illustrating the significance of context when considering mediation’s function. Therefore, it has been proposed that strategies to increase parental loyalty should take into account the unique features of each kindergarten studied; further research in other fields indicates that context influences both the magnitude and quality of mediation effects, while its setting can impact intensity as well as the nature of mediation effects (Abdolvand & Charsetad, 2013; Louis et al., 2019; Ahn et al., 2021).

These results illustrate the significance of trust between parents and kindergartens as well as brands as trust-mediating mediators, such as brands acting as mediators for social responsibility initiatives that strengthen connections between them, which ultimately increases durability and long-term viability for both parties.

This study provides insights regarding the relationship between social responsibility in kindergarten brand trust, perceived value, and loyalty to parents in the Chinese early childhood education environment. The extent to which these findings are applicable to other countries or regions needs careful consideration. Economic, cultural, and policy frameworks governing early childhood education vary between countries, having an effect on how parents view social responsibilities and the trustworthiness of educational institutions. Sweden and Finland both feature strong government oversight with significant funds dedicated to early childhood education; parents may be more attracted by institution quality and the effectiveness of curriculum than any programs for social accountability. Conversely, in areas with significant private sector involvement in early childhood education—like the United States or some Southeast Asian nations—social responsibility and branding may have more of an effect on building trust among parents.

Furthermore, differences in socioeconomic status influence the decision-making process of parents. In countries with high incomes in which parents have access to more educational options for their children, brand differentiation and trust-building mechanisms could influence enrollment decisions more so than in the developing world where accessibility and affordability remain major factors. Therefore, the basic theories that underlie this research are CSR and perceptions of value, trust, and loyalty, which could be applicable to different education settings; their value could differ depending on the local market structure as well as the policies and norms of culture. Future research will examine cross-cultural comparisons in order to confirm results across different settings, while also broadening the potential of the proposed model.

### 6.5. Recommendations

#### 6.5.1. Enhance Transparency and Communication Mechanisms

Kindergartens should publish annual social responsibility reports detailing elements like environmental protection measures, management strategies, and quality assurance of education services offered to the community. Mondi et al. (2021) recommends implementing transparency and accountability tools within education to increase parental trust in kindergarten. Transparency and accountability measures could significantly enhance educational quality as well as service provision. Additionally, kindergartens must engage in continuous communication with parents through various means—meeting with them directly, public days or online forums—quickly responding to concerns or suggestions in order to increase engagement and overall satisfaction with their service. Macy et al. (2019) emphasized that the implementation of effective evaluation methods would facilitate interactions between kindergarten teachers and parents. Transparent communication methods should include providing parents with regular updates about kindergarten developments, sharing success stories, and outlining improvement plans, all while building trust-based relationships (Farid et al., 2020). In response to complaints or feedback, kindergartens can demonstrate their dedication to problem-solving and improvement by showing that they are committed to problem-solving efforts, thereby building more loyalty among parents.

#### 6.5.2. Improving Education and Safety Standards

Maintaining an optimal teacher–student ratio and implementing sound safety protocols are key to ensuring education quality and safety. Research shows that having more professional teachers and a lower teacher–student ratio can promote better cognitive and social development in children (Wigelsworth et al., 2022; Schoch et al., 2023). Additionally, kindergartens should conduct regular safety inspections and emergency drills to ensure the safety and reliability of facilities and environments. By establishing a robust education and safety management system, kindergartens can strengthen parents’ trust in their education quality and safety standards. Kindergartens should place special emphasis on teacher development, helping educators attain highly qualified qualifications and ethics as well as an understanding of current education concepts and technologies. Research has proven the necessity of effective training and ongoing professional development to elevate preschool child education (Hau et al., 2023). Therefore, providing continuous professional development opportunities and training courses is necessary to ensure high-quality teaching and a positive environment for teacher–student interaction, effectively supporting each child’s development. Finally, by regularly evaluating and improving the educational curriculum and ensuring that teaching methods and content consistently meet high standards, kindergartens can enhance parents’ satisfaction and loyalty.

#### 6.5.3. Strengthening Cooperation Between Home and Community

Kindergartens should participate actively in organizing community services and public welfare initiatives such as environmental protection projects, charity fundraisers, and volunteer programs in their community. These efforts help to establish a positive image of the kindergarten within the community, making parents more aware of the kindergarten’s sense of social responsibility and public welfare, thereby enhancing their recognition and loyalty to the institution (Kabue et al., 2022).

Kindergartens can encourage parents to become more engaged in school life by creating parent committees, volunteer programs, and homeschool co-education programs for parents. Engaging with them increases a sense of belonging and responsibility towards the school, resulting in improved quality and creating an inviting learning environment (Sheridan et al., 2022).

Kindergartens must ensure equitable admissions of children from marginalized groups and those with special needs in order to demonstrate equality and social accountability. Family days, community-based networking activities, and parent education courses can strengthen connections between kindergarten parents and kindergarten, foster mutual support between parent groups and communities, and strengthen parent/kindergarten relationships. These channels enable parents to stay informed about kindergarten events and activities, enhancing their sense of participation and belonging (Rad et al., 2022). Cooperation and interaction within a school are vital to its success. Working together not only builds its standing within its community but also creates a more supportive and caring environment for children to grow and thrive.

#### 6.5.4. Continuous Evaluation of Parental Satisfaction

To ensure long-term loyalty with parents, kindergartens should develop continuous methods of assessing the degree of satisfaction of parents and modify their strategies in line with these assessments. Continuously run satisfaction surveys like targeted groups or structured surveys, as well as digital feedback platforms, will assist institutions in identifying the needs of parents at a moment’s notice and addressing them in a proactive way. Assessments should focus on factors like child safety, education quality, interaction between teachers and students, as well as overall confidence within an institution. By integrating evaluations into the management of kindergartens, they can better address emerging issues and modify CSR plans accordingly to meet parent expectations more efficiently.

Furthermore, data-driven decision-making methods, such as the sentiment analysis of feedback from parents and trend tracking over time, may allow kindergartens to identify patterns in parental complaints and help to improve the quality of service. Similar methods are commonly used in industries that rely on service, which depend on a constant monitoring of satisfaction. Applying these methods to early childhood education will increase parental involvement and build confidence in kindergarten operation. Future research should explore how best to incorporate continuous feedback loops in the preschool management systems to increase the satisfaction of parents and the performance of the institution.

## 7. Conclusions

This study focuses on the impact of kindergarten social responsibility on parent loyalty. By examining the two mediating variables of brand trust and parent perceived value, it reveals the complex mechanisms through which social responsibility fulfillment influences parent loyalty. The results show that kindergarten social responsibility not only directly affects parent loyalty but also indirectly influences it through brand trust and parent perceived value. The study demonstrates high reliability and validity both theoretically and empirically, further enriching the literature on social responsibility in early childhood education and providing an effective measurement method for evaluating the factors influencing kindergartens. Secondly, this study explores the formation mechanism of social responsibility fulfillment on kindergarten brand trust and parent perceived value. The results indicate that fulfilling social responsibility significantly enhances parents’ brand trust and the perceived value of kindergartens. Moreover, differences in mediation effects were observed between different types of kindergartens. In inclusive kindergartens, social responsibility partially mediates parent loyalty through brand trust. In non-inclusive kindergartens, brand trust has a more significant mediating effect between social responsibility and parent loyalty, while the direct effect is relatively small. This suggests that different types of kindergartens need to focus on different influencing paths when fulfilling their social responsibilities to effectively improve parent loyalty. Finally, this study expands the application of social responsibility within the field of preschool education and emphasizes the key role of social responsibility in kindergarten competition. Previous research on social responsibility factors has primarily focused on the corporate sector. This study empirically verifies the impact of kindergarten social responsibility on parent loyalty. The findings indicate that inclusive kindergartens are better able to win parents’ trust and enhance parent loyalty through open and transparent social responsibility practices, whereas non-inclusive kindergartens need to address their shortcomings in fulfilling social responsibilities by improving brand trust.

This study has several limitations. Firstly, the study’s sample is limited to a specific region in China, which may restrict the generalizability of the findings. Future studies should focus on cross-cultural comparisons to generalize the findings. Secondly, the reliance on self-reported data introduces potential biases, such as social desirability bias. Thirdly, the cross-sectional design limits the ability to establish causality between the variables studied. Finally, this study used a single-item measure for certain lower-level constructs, which may not fully capture their multidimensional nature. Future research should incorporate multi-item scales to enhance measurement reliability and precision. Several future works are recommended for addressing the challenges of the existing studies. Firstly, future research should explore additional mediators or moderators, such as cultural values or parental involvement, to deepen the understanding of the factors influencing parental loyalty. Secondly, examining the impact of different types of social responsibility activities on parental loyalty could identify which strategies are most effective. Finally, longitudinal studies are needed to assess the long-term effects of social responsibility on parental loyalty and how these effects may evolve over time.

## Figures and Tables

**Table 1:** Parent assessment scale for preschool education institutions.

Higher-Level Constructs	Sub-Dimensions	Question
Social responsibility	Responsibility management	Does your child’s kindergarten regularly publish social responsibility reports?
	Customer responsibilities	Does your child’s kindergarten maintain a class size and teaching staff that ensure high-quality education (teacher–student ratio does not exceed 1:7)?
		Does your child’s kindergarten have a security management mechanism for the personal information of parents and students?
		Does your child’s kindergarten have a comprehensive child safety protection mechanism and video surveillance system?
		Does your child’s kindergarten regularly survey the needs and opinions of parents and children and provide timely feedback?
	Public welfare responsibility	Does your child’s kindergarten recruit students from the general public (including special and vulnerable groups of children) without setting special admission requirements?
	Organizational responsibility	Does your child’s kindergarten comply with all national laws and regulations and operate legally?
		Does your child’s kindergarten ensure the safety and quality of drinking water, sanitation, teaching facilities, and buildings?
		Does your child’s kindergarten actively organize teachers and students to participate in green and environmental protection-themed activities to cultivate a green culture in the kindergarten?
Brand trust	Kindergarten reputation	Does the reputation of a kindergarten influence your decision on where to send your child?
		Do you have a good overall impression of your child’s kindergarten, and does it have a very good local reputation?
		Are you reassured about the teaching quality, teacher qualifications, and school conditions of your child’s kindergarten?
	Parent satisfaction	Compared to your expectations, are you very satisfied with the overall facilities, teaching environment, staff, and quality of your child’s kindergarten?
		Compared to an ideal situation, are you very satisfied with the overall facilities, teaching environment, staff, and quality of your child’s kindergarten?
		Compared to other kindergartens of the same type, are you more satisfied with the overall facilities, teaching environment, staff, and quality of your child’s kindergarten?
		In general, are you satisfied with the overall facilities, teaching environment, staff, and quality of your child’s kindergarten?
Parents perceived value	Cost-effectiveness	Does your child’s kindergarten charge reasonable fees, provide good services, and offer good value for money?
	Quality	Is your child’s kindergarten better in every aspect compared to other kindergartens with similar fee levels?
Parent loyalty	Behavioral loyalty	Has your child been attending the same kindergarten so far, and do you have no intention of transferring them to another school?
	Attitude loyalty	Are you willing to give positive feedback about your child’s kindergarten to your relatives and friends who have children and recommend it to them?

Source: developed by the authors based on survey data analysis.

**Table 2:** Results of confirmatory factor analysis fit indices for each scale in the formal survey.

Scale	Absolute Fit Index				Incremental Fit Index			
	χ^2^	χ^2^/df	GFI	RMR	RMSEA	NFI	TLI	CFI
Parent Assessment	469.590	2.863	0.937	0.025	0.050	0.924	0.941	0.949

Source: developed by the authors based on survey data analysis.

**Table 3:** Basic information of kindergarten samples (N = 745).

Demographic Variables	Group	All Kindergartens		Publicly Supported Kindergartens (N = 518)		Privately Funded Kindergarten (N = 227)	
		Frequency	Percentage (%)	Frequency	Percentage (%)	Frequency	Percentage (%)
Kinship	Father	244	32.75	186	35.9	5 8	25.6
	Mother	478	64.16	317	61.2	16 1	70.9
	Grandfather	3	0.40	1	0.2	2	0.9
	Grandmother	6	0.81	5	1.0	1	0.4
	Other	14	1.88	9	1.7	5	2.2
Education	Junior high school and below	29	3.89	19	3.7	10	4.4
	High school/technical secondary school/technical school	150	20.13	73	14.1	77	33.9
	College	141	18.93	93	18.0	48	21.1
	Undergraduate	365	48.99	287	55.4	78	34.4
	Master degree and above	60	8.05	46	8.9	14	6.2
Monthly household income	Below 5000	128	17.18	78	15.1	50	22.0
	5000–10,000	236	31.68	148	28.6	88	38.8
	10,000–30,000	296	39.73	223	43.1	73	32.2
	30,000–60,000	62	8.32	49	9.5	13	5.7
	60,000 and above	23	3.09	20	3.9	3	1.3
Monthly kindergarten fees	Below 1000	314	42.15	210	40.5	104	45.8
	1000–3000	297	39.87	218	42.1	79	34.8
	3000–5000	104	13.96	69	13.3	35	15.4
	5000–8000	23	3.09	18	3.5	5	2.2
	8000 and above	7	0.94	3	0.6	4	1.8
Kindergarten class	Kindergarten	235	31.54	153	28.1	82	36.1
	Kindergarten middle class	219	29.40	168	30.8	51	22.5
	Kindergarten small class	259	34.77	178	32.7	81	35.7
	Preschool	57	7.65	28	5.1	29	12.8
	Other	33	4.43	18	3.3	15	6.6
Kindergarten level	Provincial demonstration garden	52	6.98	43	8.3	9	4.0
	Municipal class I (Level 1) park	181	24.30	163	31.5	18	7.9
	Municipal class II (Second class) park	114	15.30	94	18.1	20	8.8
	Municipal class III (Level III) park	43	5.77	30	5.8	13	5.7
	Other or unrated	100	13.42	57	11.0	43	18.9
	Not sure	255	34.23	131	25.3	124	54.6
Location	Eastern and coastal areas	314	42.15	256	49.4	58	25.6
	Central and northern regions	305	40.94	159	30.7	146	64.3
	Western region	126	16.91	103	19.9	23	10.1

Source: developed by the authors based on survey data analysis.

**Table 4:** Parents’ evaluation scores on preschool education institutions (N = 745).

Dimensions	Index	All Kindergartens			Publicly Supported Kindergartens (N = 518)			Privately Funded Kindergartens (N = 227)		
		Score	Poor Performance (Score 3 and Below)		Score	Poor Performance (Score 3 and Below)		Score	Poor Performance (Score 3 and Below)	
			Frequency	Percentage (%)		Frequency	Percentage (%)		Frequency	Percentage (%)
Social responsibility	Responsibility management	4.0	215	28.86%	4.1	134	25.87%	3.9	81	35.68%
	Customer responsibilities		272	36.51%		188	36.29%		84	37.00%
			126	16.91%		78	15.06%		48	21.15%
			99	13.29%		75	14.48%		24	10.57%
			188	25.23%		122	23.55%		66	29.07%
	Public welfare responsibility		214	28.72%		137	26.45%		77	33.92%
	Organizational responsibility		73	9.80%		46	8.88%		27	11.89%
			68	9.13%		47	9.07%		21	9.25%
			103	13.83%		68	13.13%		35	15.42%
Brand trust	Kindergarten reputation	4.2	39	5.23%	4.2	24	4.63%	4.1	15	6.61%
			97	13.02%		56	10.81%		41	18.06%
			116	15.57%		71	13.71%		45	19.82%
	Parent satisfaction		152	20.40%		100	19.31%		52	22.91%
			160	21.48%		105	20.27%		55	24.23%
			176	23.62%		119	22.97%		57	25.11%
			104	13.96%		64	12.36%		40	17.62%
Parents perceived value	Cost-effectiveness	3.9	205	27.52%	4	119	22.97%	3.7	86	37.89%
	quality		244	32.75%		153	29.54%		91	40.09%
Parent loyalty	Behavioral loyalty	4.1	122	16.38%	4.2	78	15.06%	4.1	44	19.38%
	Loyalty		151	20.27%		105	20.27%		46	20.26%

Source: developed by the authors based on survey data analysis.

**Table 5:** Analysis of differences in parent evaluations based on different sample characteristics.

Demographic Variables	Group	Social Responsibility	Brand Trust	Parents Perceived Value	Parent Loyalty
Education	Junior high school and below	4.11	4.1 6	3.91	3.93
	High school/technical secondary school/technical school	4.21	4.2 0	4.01	4.15
	College	4.13	4. 2 7	4.05	4.26
	Undergraduate	3.94	4. 17	3.82	4.11
	Master degree and above	3.85	4. 15	3.81	4.17
F		7.705 ***	0.354	0.019 *	0.134
Monthly household income	Below 5000	4.25	4.24	4.12	4.20
	5000–10,000	4.13	4.22	3.96	4.15
	10,000–30,000	3.87	4.16	3.77	4.13
	30,000–60,000	3.96	4.17	3.85	4.05
	60000 and above	3.96	4.14	4.09	4.17
F		11.708 ***	0.724	5.074 ***	0.525
Nature of kindergarten	Inclusive kindergarten	4.07	4.23	3.99	4.18
	Non-inclusive kindergarten	3.92	4.12	3.72	4.05
F		9.685 **	6.106 *	17.955 ***	5.095 *
Kindergarten level	Provincial demonstration garden	4.01	4. 10	4.10	4.23
	Municipal class I (Level 1) Park	4.10	4.31	3.94	4.33
	Municipal class II (Second class) park	3.89	4.15	3.79	4.00
	Municipal class III (Level III) park	3.84	4. 04	3.83	3.91
	Other or Unrated	3.90	4. 02	3.69	3.97
	Not sure	4.12	4.2 1	3.99	4.17
F		4.810 ***	5.299 ***	3.305 ***	5.432 ***
Monthly kindergarten fees	Below 1000	4.14	4.26	4.11	4.22
	1000–3000	3.94	4.14	3.77	4.10
	3000–5000	3.98	4.22	3.80	4.19
	5000–8000	3.85	4.09	3.41	3.83
	8000 and above	3.81	3.65	3.43	3.29
F		5.007 ***	3.893 ***	11.082 ***	4.778 ***

Note: *, **, and *** denote statistical significance at the 10%, 5%, and 1% levels, respectively. Source: developed by the authors based on survey data analysis.

**Table 6:** Spearman correlation test.

Variables	Kinship	PE	TMHI	MKF	KN	KL	KSR	BT	PRV	PL
Kinship	1									
Parents’ education (PE)	−0.257 **	1								
Total monthly household income (TMHI)	−0.305 **	0.598 **	1							
Monthly kindergarten fees (MKF)	−0.173 **	0.350 **	0.354 **	1						
Kindergarten nature (KN)	0.080 *	−0.222 **	−0.159 **	−0.028	1					
Kindergarten level (KL)	0.234 **	−0.456 **	−0.373 **	−0.306 **	0.340 **	1				
Kindergarten social responsibility (KSR)	0.098 **	−0.170 **	−0.191 **	−0.147 **	−0.112 **	0.009	1			
Brand trust (BT)	0.035	−0.021	−0.031	−0.076 *	−0.084 *	−0.102 **	0.646 **	1		
Parents perceived value (PRV)	0.098 **	−0.118 **	−0.134 **	−0.220 **	−0.164 **	−0.018	0.591 **	0.675 **	1	
Parent loyalty (PL)	0.053	0.006	−0.033	−0.095 **	−0.088 *	−0.081 *	0.514 **	0.684 **	0.580 **	1

Note: * indicates that the correlation is significant at the 0.05 level (two-tailed); ** indicates that the correlation is significant at the 0.01 level (two-tailed). Source: developed by the authors based on survey data analysis.

**Table 7:** Pearson correlation test.

Variables	Average Value	Standard Deviation	Social Responsibility	Brand Trust	Parents Perceived Value	Parent Loyalty
Kindergarten social responsibility	4.028	0.612	1			
Brand trust	4.194	0.537	0.648 **	1		
Parents perceived value	3.905	0.798	0.580 **	0.680 **	1	
Parent loyalty	4.144	0.734	0.498 **	0.697 **	0.577 **	1

Note: ** indicates that the correlation is significant at the 0.01 level (two-tailed). Source: developed by the authors based on survey data analysis.

**Table 8:** Regression analysis between variables.

Regression Equation		Overall Fit Index			Significance of Regression Coefficient	
Outcome Variable	Predictor Variables	R	R^2^	F	β	t
Brand trust	Kindergarten social responsibility	0.663	0.439	82.234	0.583	23.103 ***
Parents perceived value	Kindergarten social responsibility	0.723	0.523	100.853	0.26 7	5.860 ***
	Brand trust				0.7 86	15.543 ***
Parent loyalty	Kindergarten social responsibility	0.714	0.510	85.121	0.104	2.349 *
	Brand trust				0.542	13.644 ***
	Parents perceived value				0.178	4.769 ***

Note: *, and *** denote statistical significance at the 10%, and 1% levels, respectively. Source: developed by the authors based on survey data analysis.

**Table 9:** Mediation of brand trust and parent perceived value between kindergarten social responsibility and parent loyalty.

Item	Effect Size	Boot Standard Error	BootCI Lower Limit	BootCI Upper Limit	Relative Mediation Effect	Proportion of Total Indirect Effect
Total indirect effect	0.551	0.030	0.401	0.518	89.74%	1
Indirect effect 1: kindergarten social responsibility → brand trust → parent loyalty	0.432	0.034	0.291	0.428	70.36%	78.40%
Indirect effect 2: kindergarten social responsibility → parents’ perceived value → parents’ loyalty	0.044	0.013	0.017	0.072	7.17%	7.99%
Indirect effect 3: kindergarten social responsibility → brand trust → parent perceived value → parent loyalty	0.075	0.018	0.028	0.098	12.21%	13.61%

Source: developed by the authors based on survey data analysis.

**Table 10:** Regression analysis between variables of publicly supported kindergartens.

Regression Equation		Overall Fit Index			Significance of Regression Coefficient	
Outcome variable	Predictor variables	R	R^2^	F	β	t
Brand trust	Social responsibility of inclusive kindergartens	0.670	0.449	69.332	0.676	19.541 ***
Parent loyalty	Social responsibility of inclusive kindergartens	0.673	0.453	60.447	0.103	2.270 *
	Brand trust				0.585	13.270 ***

Note: *, and *** denote statistical significance at the 10%, and 1% levels, respectively. Source: developed by the authors based on survey data analysis.

**Table 11:** The mediating effect of brand trust on the relationship between the social responsibility of inclusive kindergartens and parent loyalty.

Item	Effect Size	Boot	Boot CI	Boot CI	Relative Mediation Effect
		Standard Error	Lower Limit	Upper Limit	
Indirect effects	0.484	0.037	0.325	0.470	79.26%
Direct effect	0.127	0.056	0.017	0.236	2 0.74%
Total effect	0.610	0.049	0.515	0.706	

Source: developed by the authors based on survey data analysis.

**Table 12:** Regression analysis between variables of privately funded kindergartens.

Regression Equation		Overall Fit Index			Significance of Regression Coefficient	
Outcome variable	Predictor variables	R	R^2^	F	β	t
Brand trust	Social responsibility of non-inclusive kindergartens	0.644	0.415	26.060	0.632	11.965 ***
Parent loyalty	Social responsibility of non-inclusive kindergartens	0.769	0.592	45.435	0.124	2.255 *
	Brand trust				0.7 32	12.971 ***

Note: *, and *** denote statistical significance at the 10%, and 1% levels, respectively. Source: developed by the authors based on survey data analysis.

**Table 13:** The mediating effect of brand trust on the relationship between the social responsibility and parent loyalty of non-inclusive kindergartens.

Item	Effect Size	Boot	Boot CI	Boot CI	Relative Mediation Effect
		Standard Error	Lower Limit	Upper Limit	
Indirect effects	0.5 36	0.0 55	0.357	0.573	89.63%
Direct effect	0.062	0.072	0.067	0.191	10.37%
Total effect	0.598	0.068	0.465	0.732	

Source: developed by the authors based on survey data analysis.

## Data Availability

Data will be made available on request.

## References

[B1-behavsci-15-00115] Abd-El-Salam E. M. (2021). Investigating loyalty through CSR: The mediating role of brand image and brand trust. Journal of Customer Behaviour.

[B2-behavsci-15-00115] Abdolvand M., Charsetad P. (2013). Corporate social responsibility and brand equity in industrial marketing. International Journal of Academic Research in Business and Social Sciences.

[B3-behavsci-15-00115] Ahmad S., Shakir M. I., Azam A., Mahmood S., Zhang Q., Ahmad Z. (2023). The impact of CSR and green consumption on consumer satisfaction and loyalty: Moderating role of ethical beliefs. Environmental Science and Pollution Research.

[B4-behavsci-15-00115] Ahn J., Shamim A., Park J. (2021). Impacts of cruise industry corporate social responsibility reputation on customers’ loyalty: Mediating role of trust and identification. International Journal of Hospitality Management.

[B5-behavsci-15-00115] Araújo J., Pereira I. V., Santos J. D. (2023). The effect of corporate social responsibility on brand image and brand equity and its impact on consumer satisfaction. Administrative Sciences.

[B6-behavsci-15-00115] Arrive J. T., Feng M. (2018). Corporate social responsibility disclosure: Evidence from BRICS nations. Corporate Social Responsibility and Environmental Management.

[B7-behavsci-15-00115] Azmat F., Ha H. (2013). Corporate social responsibility, customer trust, and loyalty—Perspectives from a developing country. Thunderbird International Business Review.

[B8-behavsci-15-00115] Badri M. A., Mohaidat J. (2014). Antecedents of parent-based school reputation and loyalty: An international application. International Journal of Educational Management.

[B9-behavsci-15-00115] Banfield S. R., Richmond V. P., McCroskey J. C. (2006). The effect of teacher misbehaviors on teacher credibility and affect for the teacher. Communication Education.

[B10-behavsci-15-00115] Barlas A., Valakosta A., Katsionis C., Oikonomou A., Brinia V. (2023). The effect of corporate social responsibility on customer trust and loyalty. Sustainability.

[B11-behavsci-15-00115] Baron R. M., Kenny D. A. (1986). The moderator-mediator variable distinction in social psychological research. conceptual, strategic, and statistical considerations. Journal of Personality and Social Psychology.

[B12-behavsci-15-00115] Bello K. B., Jusoh A., Nor K. M. (2021). Relationships and impacts of perceived CSR, service quality, customer satisfaction and consumer rights awareness. Social Responsibility Journal.

[B13-behavsci-15-00115] Broeckelman-Post M. A., Tacconelli A., Guzmán J., Rios M., Calero B., Latif F. (2016). Teacher misbehavior and its effects on student interest and engagement. Communication Education.

[B14-behavsci-15-00115] Carvalho S. W., de Oliveira Mota M. (2010). The role of trust in creating value and student loyalty in relational exchanges between higher education institutions and their students. Journal of Marketing for Higher Education.

[B15-behavsci-15-00115] Casado-Díaz A. B., Nicolau-Gonzálbez J. L., Ruiz-Moreno F., Sellers-Rubio R. (2014). The differentiated effects of CSR actions in the service industry. Journal of Services Marketing.

[B16-behavsci-15-00115] Chen L., Fu Y., Liu Y., Wang C. (2023). The impact of logistics corporate social responsibility on supply chain performance: Using supply chain collaboration as an intermediary variable. Sustainability.

[B17-behavsci-15-00115] Cheung A. (Waikong), Pok W. C. (2019). Corporate social responsibility and provision of trade credit. Journal of Contemporary Accounting & Economics.

[B18-behavsci-15-00115] Cole G. (2017). Increasing customer loyalty: The impact of corporate social responsibility and corporate image. Annals in Social Responsibility.

[B19-behavsci-15-00115] Cullaty B. (2011). The role of parental involvement in the autonomy development of traditional-age college students. Journal of College Student Development.

[B20-behavsci-15-00115] Dereli F., Türk Kurtça T. (2023). Family engagement in early childhood education: A phenomenological study. International Journal of Psychology and Educational Studies.

[B21-behavsci-15-00115] Durkin K., Lipsey M. W., Farran D. C., Wiesen S. E. (2022). Effects of a statewide pre-kindergarten program on children’s achievement and behavior through sixth grade. Developmental Psychology.

[B22-behavsci-15-00115] Echezona O. (2024). Relationship between corporate social responsibility (CSR) initiatives and brand loyalty in emerging markets. International Journal of Business Strategies.

[B23-behavsci-15-00115] El-Kassar A., Makki D., Gonzalez-Perez M. A. (2019). Student–university identification and loyalty through social responsibility: A cross-cultural analysis. International Journal of Educational Management.

[B24-behavsci-15-00115] Farid T., Iqbal S., Khan A., Ma J., Khattak A., Din M. N. U. (2020). The impact of authentic leadership on organizational citizenship behaviors: The mediating role of affective- and cognitive-based trust. Frontiers in Psychology.

[B25-behavsci-15-00115] Fatma M., Khan I. (2023). CSR influence on brand loyalty in banking: The role of brand credibility and brand identification. Sustainability.

[B26-behavsci-15-00115] Green T., Peloza J. (2011). How does corporate social responsibility create value for consumers?. Journal of Consumer Marketing.

[B27-behavsci-15-00115] Greenberg M. T. (2023). Evidence for social and emotional learning in schools.

[B28-behavsci-15-00115] Han H., Yu J., Lee K., Baek H. (2020). Impact of corporate social responsibilities on customer responses and brand choices. Journal of Travel & Tourism Marketing.

[B29-behavsci-15-00115] Harman H. H. (1968). Modern factor analysis.

[B30-behavsci-15-00115] Hau H. G., Selenius H., Bjorck E. (2023). Exploring Swedish preschool teachers’ perspectives on applying a self-reflection tool for improving inclusion in early childhood education and Care. Frontiers in Education.

[B31-behavsci-15-00115] Hayes A. F. (2022). Introduction to mediation, moderation, and conditional process analysis: A regression-based approach.

[B32-behavsci-15-00115] He Y., Lai K. K. (2014). The effect of corporate social responsibility on brand loyalty: The mediating role of brand image. Total Quality Management & Business Excellence.

[B33-behavsci-15-00115] Hu B. Y., Li Y., Wang C., Wu H., Vitiello G. (2021). Preschool teachers’ self-efficacy, classroom process quality, and children’s social skills: A Multilevel Mediation Analysis. Early Childhood Research Quarterly.

[B34-behavsci-15-00115] Huai J. (2024). Working together: Prioritizing education development and transformation for a better future.

[B35-behavsci-15-00115] Huo C., Hameed J., Zhang M., Ali A. F. B. M., Hashim N. A. A. N. (2022). Modeling the impact of corporate social responsibility on sustainable purchase intentions: Insights into brand trust and brand loyalty. Economic Research-Ekonomska Istraživanja.

[B36-behavsci-15-00115] Islam T., Islam R., Pitafi A. H., Xiaobei L., Rehmani M., Irfan M., Mubarak M. S. (2021). The impact of corporate social responsibility on customer loyalty: The mediating role of corporate reputation, customer satisfaction, and trust. Sustainable Production and Consumption.

[B37-behavsci-15-00115] Jalilvand M. R., Vosta L. N., Mahyari H. K., Pool J. K. (2017). Social responsibility influence on customer trust in hotels: Mediating effects of reputation and word-of-mouth. Tourism Review.

[B38-behavsci-15-00115] Jin Y., Park S., Yoo J. (2017). Effects of corporate social responsibility on consumer credibility perception and attitude toward luxury brands. Social Behavior and Personality.

[B39-behavsci-15-00115] Joo S., Miller E. G., Fink J. S. (2019). Consumer evaluations of CSR authenticity: Development and validation of a multidimensional csr authenticity scale. Journal of Business Research.

[B40-behavsci-15-00115] Kabue M., Abubakar A., Ssewanyana D., Angwenyi V., Marangu J., Njoroge E., Ombech E., Mokaya M. M., Obulemire E. K., Mugo C., Malti T., Moran G., Martin M., Proulx K., Marfo K., Zhang L., Lye S. (2022). A community engagement approach for an integrated early childhood development intervention: A case study of an urban informal settlement with kenyans and embedded refugees. BMC Public Health.

[B41-behavsci-15-00115] Kim M., Yin X., Lee G. (2020). The effect of CSR on corporate image, customer citizenship behaviors, and customers’ long-term relationship orientation. International Journal of Hospitality Management.

[B42-behavsci-15-00115] Kock F., Berbekova A., Assaf A. G. (2021). Understanding and managing the threat of common method bias: Detection, prevention and control. Tourism Management.

[B43-behavsci-15-00115] Konuk F. A. (2018). The role of store image, perceived quality, trust and perceived value in predicting consumers’ purchase intentions towards organic private label food. Journal of Retailing and Consumer Services.

[B44-behavsci-15-00115] Lacap J. P. G., Cham T. H., Lim X. J. (2021). The influence of corporate social responsibility on brand loyalty and the mediating effects of brand satisfaction and perceived quality. International Journal of Economics & Management.

[B45-behavsci-15-00115] Latif K. F., Pérez A., Sahibzada U. F. (2020). Corporate Social Responsibility (CSR) and customer loyalty in the hotel industry: A cross-country study. International Journal of Hospitality Management.

[B46-behavsci-15-00115] Le T. T., Huan N. Q., Hong T. T. T., Tran D. K. (2021). The contribution of corporate social responsibility on SMEs performance in emerging country. Journal of Cleaner Production.

[B47-behavsci-15-00115] Leclercq-Machado L., Alvarez-Risco A., Esquerre-Botton S., Almanza-Cruz C., de las Mercedes Anderson-Seminario M., Del-Aguila-Arcentales S., Yáñez J. A. (2022). Effect of corporate social responsibility on consumer satisfaction and consumer loyalty of private banking companies in Peru. Sustainability.

[B48-behavsci-15-00115] Lee S., Han H., Radic A., Tariq B. (2020). Corporate Social Responsibility (CSR) as a customer satisfaction and retention strategy in the chain restaurant sector. Journal of Hospitality and Tourism Management.

[B49-behavsci-15-00115] Lee Y.-C., Wang Y.-C., Lu S.-C., Hsieh Y.-F., Chien C.-H., Tsai S.-B., Dong W. (2016). An empirical research on customer satisfaction study: A consideration of different levels of performance. SpringerPlus.

[B50-behavsci-15-00115] Li X., Zhang X., Zhao Y., Zhang L., Shang J. (2023). Exploring the role of learning through play in promoting multimodal learning among children: A pilot study in Chinese first-tier cities. Frontiers in Psychology.

[B51-behavsci-15-00115] Liu Y., Chen Y., Ren Y., Jin B. (2021). Impact mechanism of corporate social responsibility on sustainable technological innovation performance from the perspective of corporate social capital. Journal of Cleaner Production.

[B52-behavsci-15-00115] Louis D., Lombart C., Durif F. (2019). Impact of a retailer’s CSR activities on consumers’ loyalty. International Journal of Retail & Distribution Management.

[B53-behavsci-15-00115] Lu J., Ren L., Zhang C., Wang C., Shahid Z., Streimikis J. (2020). The influence of a firm’s CSR initiatives on brand loyalty and brand image. Journal of Competitiveness.

[B54-behavsci-15-00115] Lv Y., Wu M., Ma C., Hao X., Zeng X. (2022). Assessment of the status quo of social responsibility performance of inclusive kindergartens: Evidence from China. PLoS ONE.

[B55-behavsci-15-00115] Macy M., Bagnato S., Weiszhaupt K. (2019). Family-friendly communication via authentic assessment for early childhood intervention programs. Zero to Three.

[B56-behavsci-15-00115] Mahmud Z., Ibrahim H., Amat S., Salleh A. (2011). Family communication, sibling position and adolescents’ sense of responsibility. World Applied Sciences Journal.

[B57-behavsci-15-00115] Mann S. C., Islam T. (2015). The roles and involvement of local government human resource professionals in coastal cities emergency planning. Journal of Homeland Security and Emergency Management.

[B58-behavsci-15-00115] Markovic S., Iglesias O., Qiu Y., Bagherzadeh M. (2022). The CSR imperative: How CSR influences word-of-mouth considering the roles of authenticity and alternative attractiveness. Business & Society.

[B59-behavsci-15-00115] Mitnick B. M., Windsor D., Wood D. J. (2023). Moral CSR. Business & Society.

[B60-behavsci-15-00115] Mittal V., Han K., Frennea C., Blut M., Shaik M., Bosukonda N., Sridhar S. (2023). Customer satisfaction, loyalty behaviors, and firm financial performance: What 40 years of research tells us. Marketing Letters.

[B61-behavsci-15-00115] MOE (2023). China national basic situation of various regions.

[B62-behavsci-15-00115] Mondi C. F., Giovanelli A., Reynolds A. J. (2021). Fostering socio-emotional learning through early childhood intervention. International Journal of Child Care and Education Policy.

[B63-behavsci-15-00115] Mostafa R. B., Hamieh L. (2022). How CSR activities affect student attitudinal and behavioral loyalty in the lebanese educational sector?. International Journal of Customer Relationship Marketing and Management.

[B64-behavsci-15-00115] Muskat B., Hörtnagl T., Prayag G., Wagner S. (2019). Perceived quality, authenticity, and price in tourists’ dining experiences: Testing competing models of satisfaction and behavioral intentions. Journal of Vacation Marketing.

[B65-behavsci-15-00115] Na M., Rong L., Ali M. H., Alam S. S., Masukujjaman M., Ali K. A. M. (2023). The mediating role of brand trust and brand love between brand experience and loyalty: A study on smartphones in China. Behavioral Sciences.

[B66-behavsci-15-00115] Park E., Kim K. J. (2019). What drives “customer loyalty”? The role of corporate social responsibility. Sustainable Development.

[B67-behavsci-15-00115] Park J., Lee H., Kim C. (2014). Corporate social responsibilities, consumer trust and corporate reputation: South Korean consumers’ perspectives. Journal of Business Research.

[B68-behavsci-15-00115] Pfajfar G., Shoham A., Małecka A., Zalaznik M. (2022). Value of corporate social responsibility for multiple stakeholders and social impact—relationship marketing perspective. Journal of Business Research.

[B69-behavsci-15-00115] Pivato S., Misani N., Tencati A. (2008). The impact of corporate social responsibility on consumer trust: The case of organic food. Business Ethics: A European Review.

[B70-behavsci-15-00115] Rad D., Redeş A., Roman A., Ignat S., Lile R., Demeter E., Egerău A., Dughi T., Balaş E., Maier R., Kiss C., Torkos H., Rad G. (2022). Pathways to inclusive and equitable quality early childhood education for achieving SDG4 Goal—A scoping review. Frontiers in Psychology.

[B71-behavsci-15-00115] Rao N., Yang Y., Su Y., Cohrssen C. (2023). Promoting equity in access to quality early childhood education in China. Children.

[B72-behavsci-15-00115] Rasoolimanesh S. M., Shafaei A., Nejati M., Tan P. L. (2023). Corporate social responsibility and international students mobility in higher education. Social Responsibility Journal.

[B73-behavsci-15-00115] Safeer A. A., Liu H. (2023). Role of corporate social responsibility authenticity in developing perceived brand loyalty: A consumer perceptions paradigm. Journal of Product & Brand Management.

[B74-behavsci-15-00115] Schoch A. D., Gerson C. S., Halle T., Bredeson M. (2023). Children’s learning and development benefits from high-quality early care and education: A summary of the evidence.

[B75-behavsci-15-00115] Servera-Francés D., Piqueras-Tomás L. (2019). The effects of corporate social responsibility on consumer loyalty through consumer perceived value. Economic Research-Ekonomska Istrazivanja.

[B76-behavsci-15-00115] Sheridan S. M., Knoche L. L., Boise C. (2022). Getting ready: A relationship-based approach to parent engagement in early childhood education settings. Family-school partnerships during the early school years.

[B77-behavsci-15-00115] Solikhah M., Amelia A. (2023). The influence of brand awareness, perceived quality, brand association, brand loyalty and price on Daihatsu car buying decisions (case study at Daihatsu pemalang car dealership). Journal Research of Social Science, Economics, and Management.

[B78-behavsci-15-00115] Tamanna T. (2024). The mediating role of brand commitment in the relationship between brand trust and brand loyalty. Lecture Notes in Networks and Systems.

[B79-behavsci-15-00115] Villagra N., Monfort A., Herrera J. S. (2021). The mediating role of brand trust in the relationship between brand personality and brand loyalty. Journal of Consumer Behaviour.

[B80-behavsci-15-00115] Vlachos P. A., Tsamakos A., Vrechopoulos A. P., Avramidis P. K. (2009). Corporate social responsibility: Attributions, loyalty, and the mediating role of trust. Journal of the Academy of Marketing Science.

[B81-behavsci-15-00115] Wang Z., Xu G. (2017). Research on the enterprise value evaluation model embedded with CSR behavior. East China Economic Management (Chinese).

[B82-behavsci-15-00115] Wei A.-P., Peng C.-L., Huang H.-C., Yeh S.-P. (2020). Effects of corporate social responsibility on firm performance: Does customer satisfaction matter?. Sustainability.

[B83-behavsci-15-00115] Wigelsworth M., Verity L., Mason C., Qualter P., Humphrey N. (2022). Social and emotional learning in primary schools: A review of the current state of evidence. British Journal of Educational Psychology.

[B84-behavsci-15-00115] Yang D., Chen P., Wang K., Li Z., Zhang C., Huang R. (2023). Parental involvement and student engagement: A review of the literature. Sustainability.

[B85-behavsci-15-00115] Yildiz S., Kilic G. N., Acar I. H. (2023). Early childhood education during the COVID-19 outbreak: The perceived changing roles of preschool administrators, teachers, and parents. Early Childhood Education Journal.

[B86-behavsci-15-00115] Zhang N. (2022). How does CSR of food company affect customer loyalty in the context of COVID-19: A moderated mediation model. International Journal of Corporate Social Responsibility.

